# Dilute-As AlNAs Alloy for Deep-Ultraviolet Emitter

**DOI:** 10.1038/srep22215

**Published:** 2016-02-24

**Authors:** Chee-Keong Tan, Damir Borovac, Wei Sun, Nelson Tansu

**Affiliations:** 1 Center for Photonics and Nanoelectronics, Department of Electrical and Computer Engineering, Lehigh University, Bethlehem, PA 18015, USA

## Abstract

The band structures of dilute-As AlNAs alloys with As composition ranging from 0% up to 12.5% are studied by using First-Principle Density Functional Theory (DFT) calculation. The energy band gap shows remarkable reduction from 6.19 eV to 3.87 eV with small amount of As content in the AlNAs alloy, which covers the deep ultraviolet (UV) spectral regime. A giant bowing parameter of 30.5 eV ± 0.5 eV for AlNAs alloy is obtained. In addition, our analysis shows that the crossover between crystal field split-off (CH) band and heavy hole (HH) bands occurs in the dilute-As AlNAs alloy with As-content of ~1.5%. This result implies the possibility of dominant transverse electric (TE)-polarized emission by using AlNAs alloy with dilute amount of As-content. Our findings indicate the potential of dilute-As AlNAs alloy as the new active region material for TE-polarized III-Nitride-based deep UV light emitters.

Group III-Nitride semiconductor is widely regarded as the important material class for solid-state lighting, medical and energy efficiency technologies^1^,^2^,^3^,^4^,^5^,^6^,^7^,^8^,^9^,^10^,^11^,^12^,^13^,^14^,^15^,^16^. The importance of III-Nitride semiconductor material such as AlGaInN alloy is attributed to the access of broad coverage in ultraviolet (UV), visible and infrared spectrum. The advances in the optoelectronic applications such as light emitting diodes (LEDs) and laser diodes are made possible by the rapid development of III-Nitride materials. Moreover, the key contributions on III-Nitride based LEDs have recently been awarded by the Nobel Prize in Physics 2014^17^.

Extensive studies carried out in the past decade in III-Nitride emitters are based on InGaN, AlGaN and AlInN alloys for visible and deep UV light emissions. In contrast, the progress of developing mixed-anion III-Nitride alloys is relatively slow and the development is still in the early stage. There are significant studies in other mixed anion material systems^18^,^19^,^20^,^21^,^22^,^23^,^24^. Specifically, the incorporation of N impurity in the GaAs material has led to the advances in the field of dilute-N InGaAs(N)-based alloy, resulting in the state-of-the-art and low threshold laser devices for telecommunication applications^18^,^19^,^20^. These advances can only be realized through significant understanding in the electronic properties and the device physics, as well as the improvement in material epitaxy^18^,^19^,^20^,^21^,^22^,^23^.

Our recent work has shown the promising potential of the dilute-As GaNAs alloy as an alternative light emitting active material for high efficiency visible light applications^25^,^26^,^27^. Specifically, the understanding of the electronic properties of the dilute-As GaNAs alloys revealed the ability of the alloy in suppressing the detrimental interband Auger recombination effect^25^,^26^. While dilute-As GaNAs alloy shows promising progress from the experimental and theoretical aspects for visible spectral regime^3^,^20^,^21^,^22^,^23^,^24^,^25^,^26^,^27^,^28^,^29^,^30^,^31^,^32^, the effect of arsenic (As) incorporation in other nitride-based materials such as AlN alloy for deep UV light emitters is still unexplored. Deep UV light emitters have become increasingly important in recent years^8^,^33^,^34^,^35^,^36^,^37^,^38^,^39^,^40^, and the pursuit of novel materials, such as dilute-As AlNAs alloy, can further stimulate the development of the UV light-based technology. Up to date the literature on AlNAs alloy is extremely limited^28^. Hence the understanding of the electronic properties of dilute-As AlNAs alloy is an important first step towards developing the alloy for deep UV applications.

In this work, we present the electronic properties of dilute-As AlNAs alloy by using First-Principle Density Functional Theory (DFT). The arsenic (As) atoms are introduced into the AlN bulk material to form the AlNAs ternary alloy with As-content ranging from 0% up to 6.25%. The DFT-calculated band structures and the related electronic properties including energy band gap and effective masses of dilute-As AlNAs alloy are presented. In addition, the valence band structures of dilute-As AlNAs alloys are further investigated, in which the effect of As impurity onto the issue of band crossover between the crystal-field split-off (CH) and heavy hole (HH) bands is briefly discussed.

## Computational Method

**Figure 1:**
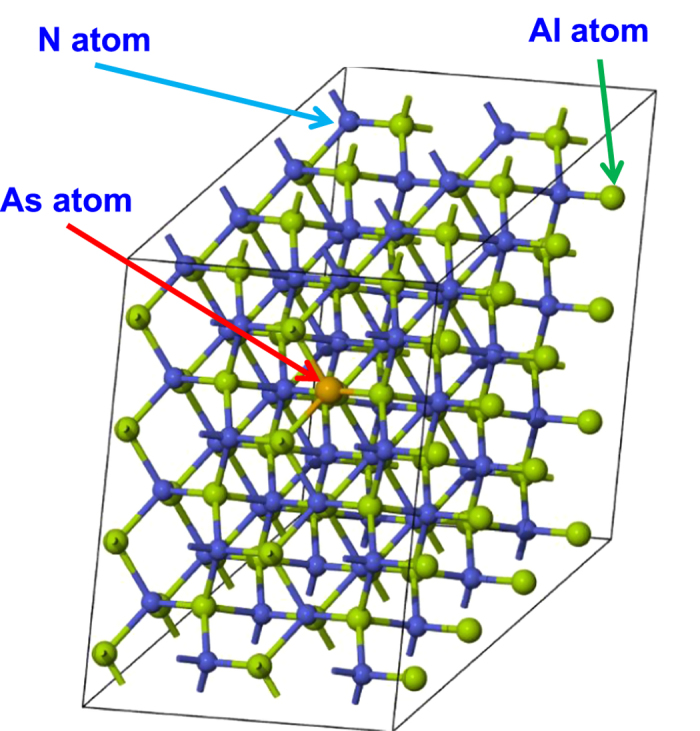
Illustration of 4 × 4 × 2 supercell with 128 atoms built using MedeA-VASP software. This 128-atom supercell consists of 64 Aluminium (Al) atoms, 63 Nitrogen (N) atoms and 1 arsenic (As) atom which corresponds to 1.56%-As in AlNAs alloy.

In our DFT analysis, the supercell approach has been employed to build the corresponding crystal structures for AlNAs alloy for the band calculations. Figure 1(a) illustrates a constructed 4 × 4 × 2 AlNAs supercell with a total of 128 atoms, which consists of 64 Al atoms, 63 N atoms and 1 substituted As atom. The illustrated 128-atom supercell corresponds to 1.56%-As in the dilute-As AlNAs alloy. The supercell size was varied in our calculations to provide different As percentage for the AlNAs alloy, which is similar to previous work^25^. Note that in each AlNAs supercell only one N atom is replaced by the As atom. The band structure calculations for AlNAs alloy were performed using the projector augmented wave (PAW) method as implemented in the MedeA-VASP software^42^. Local density approximation (LDA) exchange-correlation potential was employed in our calculations, and the electronic wave functions are described in plane wave basis with a cutoff energy of 400 eV. The structure optimization was performed for the supercell by relaxing the atom positions with the Hellmann-Feynman force set to 0.02 eV/Å. The external stress in the supercell was set to 0 GPa, and the energy convergence tolerance was set to 1 × 10^−5^ eV/atom. Different Monkhorst-Pack k-point meshes were generated in the calculations attributed to the use of different supercell sizes. In addition, the spin-orbit coupling effect is not considered in the DFT calculation due to its insignificant effect in the wide band gap III-Nitride semiconductor alloy.

## Band Parameters of Dilute-As AlNAs Alloy

**Figure 2:**
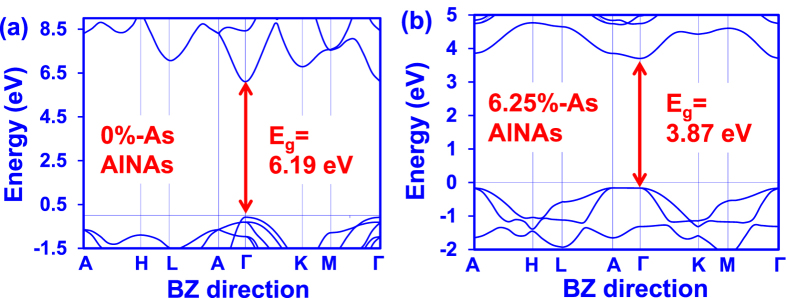
DFT-calculated band structures of AlNAs alloys with (**a**) 0% and (**b**) 6.25% As-content, with the energy band gap (E_g_) taken as the energy difference between the CBM and VBM.

Figure 2(a,b) present the DFT-calculated band structures for AlN and AlN_0.9375_As_0.0625_ alloys respectively. As shown in Fig. 2, the incorporation of As atoms in the AlN alloy has shown to affect both the conduction and valence bands, leading to significant difference in the band dispersions between the AlN and AlNAs alloys. The effect of the As-related defect in the band structures of AlN alloy is similar to that of the dilute-As GaNAs alloy^25^, in which the defect states are introduced in the energy gap resulting in the band structure modifications. Therefore, the incorporation of As impurity in the AlN alloy to form the AlNAs alloy is expected to lead to significant changes in the optoelectronic properties of the alloy. Meanwhile, as shown in Fig. 2, the conduction band minimum (CBM) and the valence band maximum (VBM) for dilute-As AlNAs alloys are located at the gamma point (Γ) in the Brillouin Zone. The direct band gap property of dilute-As AlNAs alloys indicates the potential of the alloys to achieve high efficiency electron-hole recombination, a key ingredient for photonics device applications.

The energy band gap value for the dilute-As AlNAs alloy has been taken as the energy difference between the CBM and VBM of the band structures. Note that the scissor operator has been applied in our analysis for the dilute-As AlNAs alloys in order to correct the energy band gap error originated from the LDA calculations^43^. As the scissor operator is inversely proportional to the dielectric constant, the energy correction from scissor operator ranges from 1.8 eV for AlNAs alloy with 6.25% As-content to 1.98 eV for AlN alloy, attributed to the linear interpolation of dielectric constant between AlN and AlAs alloy. As shown in Fig. 2, the energy band gap reduces significantly from 6.19 eV to 3.87 eV with the incorporation of 6.25% As concentrations into the AlN alloy. The large reduction of 2.32 eV in the energy band gap with dilute amount of As impurity (6.25%-As) in the AlN alloy indicates the significant band gap narrowing effect. Similar band gap narrowing effect has also been reported in other dilute-anion III-Nitride material system^3^,^25^,^29^,^30^,^31^. Specifically, dramatic reduction of the band gap energy of the host material has been observed in the dilute-As GaNAs material, which provides the possibility of accessing the entire visible spectral range from blue to red emission^25^. Note that the replacement of a single N atom with As atom in the GaN results in the introduction of defect states in the GaN energy gap^22^. Similar behaviour is expected in the AlN alloy when an N atom is replaced with an As atom, in which the defect states introduced in the energy gap lead to strong modification of the AlN band structure. Thus, the optoelectronic properties of the AlN alloy are heavily affected, leading to unusual behaviour in the AlNAs alloy. Interestingly, the alloys of AlN such as the AlInN alloy also display unusual behaviour attributed to the formation of In-related localized states^41^.

**Figure 3:**
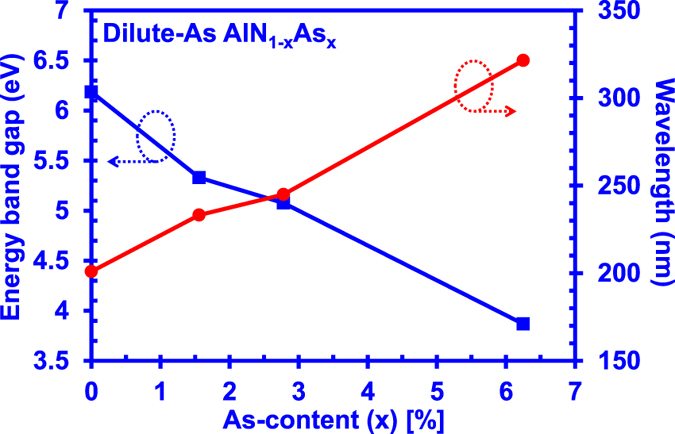
DFT-calculated energy band gap and corresponding emission wavelength of dilute-As AlNAs alloys with As-content varying from 0% up to 6.25%.

Figure 3 presents the DFT-calculated energy band gaps and the corresponding emission wavelength for dilute-As AlNAs alloys with As-content from 0% up to 6.25%. The solid line in Fig. 3 is to show a general trend of energy band gap reduction for the dilute-As AlNAs alloy. As shown in Fig. 3, the energy band gap reduces as the As-content increases from 0% to 6.25% in the AlN alloy, while the emission wavelength of the dilute-As AlNAs alloy covers the regime from 200 nm up to 320 nm. The broad wavelength coverage indicates the accessibility of the dilute-As AlNAs alloy to the entire deep UV spectral regime, which shows the potential of the alloy as a candidate for the deep UV optoelectronic device applications. Previous DFT work in the dilute-As GaNAs alloy has shown a trend of energy band gap reduction similar to the experimental data^25^, The trend of energy band gap reduction in the dilute-As AlNAs alloy is expected to be similar to that of dilute-As GaNAs alloy, albeit the current analysis in this work is still in the early stage. Note that there is no comparison available for the experimental data since the dilute-As AlNAs alloy is yet to be understood prior to this study.

**Figure 4:**
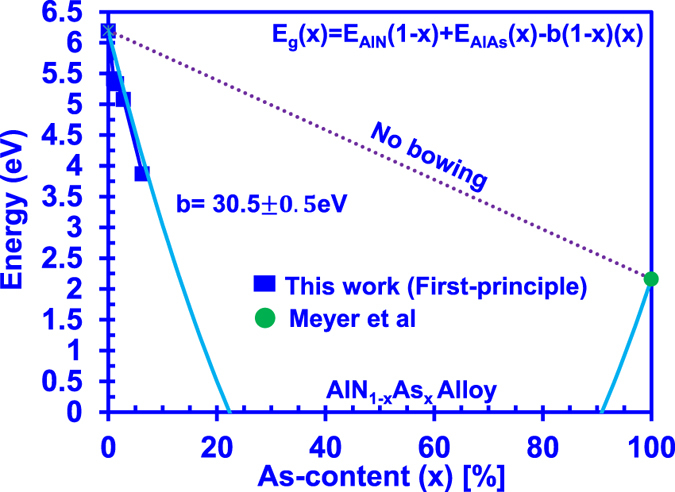
Energy band gap of AlNAs alloy with full composition range with corresponding bowing parameter obtained through line fitting with the data.

Figure 4 shows the energy band gap of AlNAs alloy with full composition range. The energy band gap for AlNAs alloys can be described using the following equation E_g_(x) = E_AlN_(1 − x) + E_AlAs_(x)–b(1 − x)(x), where b is the bowing parameter. By line fitting the equation with the DFT-calculated band gap data for dilute-As AlNAs alloy, our analysis reveals a giant bowing parameter of 30.5 eV ± 0.5 eV, as shown in Fig. 4. The findings of large bowing parameters have also been reported in other mixed-anion material systems including dilute-nitride-based materials^21^,^22^,^23^ and dilute-As GaNAs alloy^25^. The unusually large bowing parameter in AlNAs alloy can be attributed to the large atomic size difference between N atom and As atom. It is important to note that the bowing parameter is strongly composition dependent in highly-mismatched alloy such as the GaAsN alloy^21^,^22^,^23^, and similar behaviour is also expected in AlNAs alloy when the As-content becomes higher than 10%. The single bowing parameter provided in this work provides a suitable fit to the corresponding band gap energy of dilute-As AlNAs alloy with As-content less than 6.25%. The literature on AlNAs alloy is still extremely limited up to present^28^,^44^. Thus additional studies are still required to provide further understanding of this AlNAs alloy, as well as the composition dependency of the bowing parameter in the alloy.

**Figure 5:**
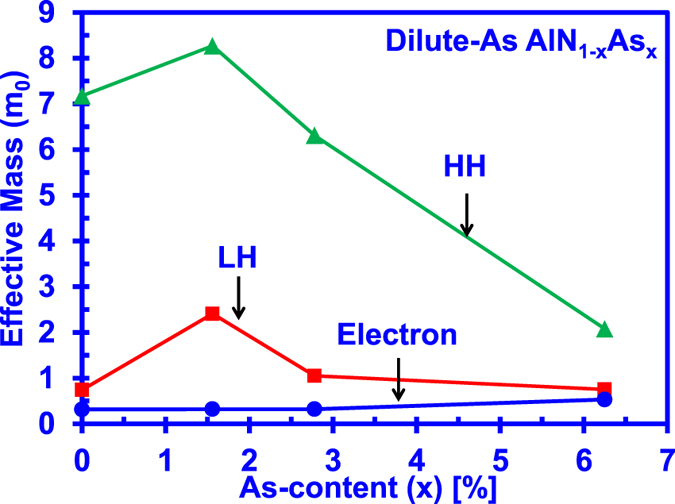
Comparison of effective masses for electron, heavy hole (HH) and light hole (LH) that are obtained through energy dispersion relation and parabolic line fitting with the DFT-calculated band structures for dilute-As AlNAs alloy.

Figure 5 shows the carrier effective masses of dilute-As AlNAs alloy for As-content from 0% up to 6.25%. The effective mass values for the dilute-As AlNAs alloys are obtained by employing parabolic line fitting method to fit the DFT-calculated band structures^45^. The average effective masses are calculated taking into account the effective masses in parallel and perpendicular directions near the gamma (Γ) point. The effective masses are calculated by fitting the calculated energy band dispersions, in which the effective masses calculated for AlN alloy are in agreement with previous literature^45^. For the AlNAs alloy, the electron effective mass is presented in Fig. 5 and it can be observed that the average effective mass of the electron increases slightly, with increasing As-content. On the other hand, the incorporation of As concentrations into the AlN alloy leads to significant changes in the average effective masses for both heavy holes and light holes. The phenomena observed in our findings are similar with the corresponding characteristics shown in dilute-As GaNAs alloy, in which there are significant changes in the average effective masses for both heavy holes and light holes in the GaNAs material. The phenomena behind this significant hole effective mass changes is attributed to the strong valence bands modifications with the As impurity incorporation into the AlN alloy^25^.

## Valence Band Crossover in Dilute-As AlNAs Alloy

The band gap coverage provided by dilute-As AlNAs alloy is attractive for deep UV emitting applications. Up to present, the state-of-the-art deep UV III-Nitride based light emitters have been employing AlGaN ternary alloy as the active region^35^,^36^. One of the challenging issues in the AlGaN-based deep-UV light emitter is the fundamental valence band crossover issue, in which the crystal-field split-off band (CH) is on top of the heavy hole (HH) band and light hole (LH) band in the high Al-content AlGaN material^37^. This leads to dominant transverse-magnetic (TM) polarized emission which is attributed to the transition between the conduction band and the CH band in the AlGaN quantum well^37^. TM-polarized emission is undesirable for the deep UV light emitter as the light propagation parallel to the quantum well plane is difficult for light extraction^38^. The crossover of CH and HH/LH band will only occur when the Al-content becomes sufficiently small (<60%) for the AlGaN material, resulting in dominant TE-polarized emission that is attributed to the C-HH transition^37^. Overcoming the valence band crossover issue is thus critical to achieve dominant TE-polarized emission for the deep UV emitters.

**Figure 6:**
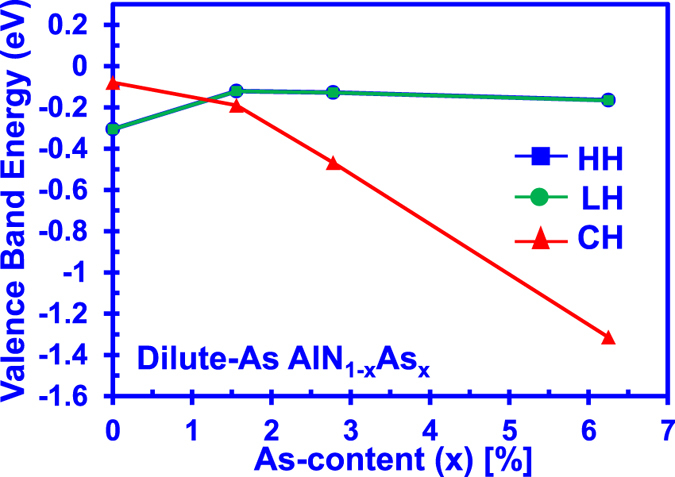
Valence band edges of the heavy hole (HH), light hole (LH) and crystal-field split-off (CH) bands for the dilute-As AlNAs alloys with As-content varying from 0% up to 6.25%. Note that the lines in the figure are provided only as guides to represent the trend of how the energy splitting between CH and HH band varies as a function of As-content in the alloy.

Figure 6 presents the valence energy band edges (HH, LH, and CH) as a function of As-content for dilute-As AlNAs alloys with As-content varying from 0% up to 6.25%. Note that the lines in Fig. 6 are provided only as guides to represent the trend of how the energy splitting between CH and HH band varies as a function of As-content in the dilute-As AlNAs alloy. As shown in Fig. 6, the CH band is on top of the HH/LH bands in the AlN alloy as expected. On the other hand, the HH/LH bands sit above CH band for the dilute-As AlNAs alloy with 1.56% As-content. Thus the crossover between the HH/LH and CH bands is estimated to occur with As-content of ~1.5% in the AlNAs alloy. Further studies will be required to predict a more accurate band crossover composition for the AlNAs alloy. Following the increase in the As-content of the dilute-As AlNAs alloy, the energy separation between HH/LH bands and CH band further increases. The phenomenon of the increasing energy splitting between HH/LH bands and CH band with the increasing anion impurity composition has also been observed in other highly-mismatched alloy such as the GaAsBi alloy^24^. This phenomenon in the AlNAs alloy is partially caused by the increase in the anion atomic number^46^. On the other hand, this phenomenon might be affected by the clustering of atoms which in turn has strong effects on the band structure as observed in the InGaN alloy^15^,^16^. However, the clustering effect is not taken into consideration in this work since only one N atom is replaced in each AlNAs supercell. Further investigations will be needed to provide a more thorough discussion for the phenomenon of the increasing energy splitting in the AlNAs alloy. In addition, the large energy separation between CH band energy and HH/LH band energy for the dilute-As AlNAs alloy is important to avoid the valence bands cluttering effect shown in AlGaN material^37^.

Our findings indicate the possibility of using dilute-As AlNAs alloy to achieve dominant transition between conduction band and HH/LH bands instead of conduction and CH bands. The possibility of using only dilute amount of As impurity in the AlN alloy to overcome the valence band crossover issue shows the strong potential of dilute-As AlNAs alloy to achieve dominant TE-polarized emission. The field of dilute-As AlNAs is still in the extremely early stage due to the novelty of this material. Our goal in this work is to open up a new direction and enhance the understanding in the dilute-As AlNAs material for the UV emission. The identification of the dilute-As AlNAs alloy as a promising active material for deep UV emitters, as well as the identification of overcoming valence band crossover issue using this alloy, will provide a clear and strong motivation on the importance of the pursuit of this material system. Future studies in both the experimental and theoretical aspects are required to provide further understanding in the dilute-As AlNAs material.

## Conclusion

In summary, the band structures of dilute-As AlNAs alloys from 0%-As up to 6.25%-As were calculated using First-Principle Density Functional Theory calculation. The band structures of the dilute-As AlNAs alloys are presented, which shows the significant reduction of the energy band gap with the incorporation of As-content into the AlN alloy. Specifically, the band gap energy of dilute-As AlNAs alloy ranges from 6.19 eV to 3.87 eV with As-content varying from 0% to 6.25% respectively. The broad band gap energy coverage of the dilute-As AlNAs material with dilute amount of As concentrations implies the accessibility of emission wavelength range from 200 nm to 320 nm, which is the entire deep UV spectral regime. The band gap bowing and carrier effective masses parameters for the dilute-As AlNAs alloy are obtained through line fitting with the DFT-calculated band structures of the alloys. The band properties show similar characteristics as observed in the dilute-As GaNAs alloys, specifically on the remarkable band gap narrowing effect provided by the As incorporation into the host material. In addition, our findings show that the HH and LH bands crossover with the CH band in the dilute-As AlNAs alloy with As-content of ~1.5%, which indicate the possibility of achieving dominant TE-polarized emission with the alloy. The understanding of the electronic properties of the dilute-As AlNAs semiconductor as presented in this work will provide a clear motivation to pursue this alloy for deep UV III-Nitride optoelectronic applications. Our finding shows that the addition of a minute amount of As into AlN to form dilute-As AlNAs results in a dramatic change in its corresponding electronic band structure property, which opens a new avenue of using this alloy to form heterostructure serving as active regions for photonics devices in the deep UV and mid UV spectral regime.

## Additional Information

**How to cite this article**: Tan, C.-K. *et al.* Dilute-As AlNAs Alloy for Deep-Ultraviolet Emitter. *Sci. Rep.* **6**, 22215; doi: 10.1038/srep22215 (2016).
